# Does groundwater protection in Europe require new EU-wide environmental quality standards?

**DOI:** 10.3389/fchem.2014.00032

**Published:** 2014-06-02

**Authors:** Matteo Balderacchi, Maria Filippini, Alexandra Gemitzi, Björn Klöve, Marco Petitta, Marco Trevisan, Przemysław Wachniew, Stanisław Witczak, Alessandro Gargini

**Affiliations:** ^1^Istituto di Chimica Agraria e Ambientale, Università Cattolica del Sacro CuorePiacenza, Italy; ^2^Biological, Geological and Environmental Sciences Department, Alma Mater Studiorum University of BolognaBologna, Italy; ^3^Department of Environmental Engineering, Democritus University of ThraceXanthi, Greece; ^4^Water Resources and Environmental Engineering Laboratory, University of OuluOulu, Finland; ^5^Dipartimento di Scienze della Terra, Università La Sapienza di RomaRoma, Italy; ^6^Faculty of Physics and Applied Computer Science, AGH University of Science and TechnologyKrakow, Poland; ^7^Faculty of Geology, Geophysics and Environmental Protection, AGH University of Science and TechnologyKrakow, Poland

**Keywords:** groundwater monitoring, GWD, pesticide, nitrates, OWC, conceptual modeling, annex I, annex II

## Abstract

The European Groundwater Directive could be improved by limiting the scopes of the Annexes I and II to the manmade and natural substances, respectively, and by defining a common monitoring protocol. The changes in the European landuse patterns, in particular the urban sprawl phenomena, obscure the distinction between the point and diffuse sources of contamination. In the future more importance will be given to the household contamination. Moreover, the agricultural environment could be used for developing new conceptual models related to the pharmaceuticals.

## Introduction

Groundwater is a source of fresh water for human consumption, irrigation and ecosystem needs, and its protection is a key environmental objective. Its pollution by anthropogenic activities is a threat to human health and wellbeing and to dependent ecosystems. The European Union has promoted several directives for protecting groundwater from pollution and deterioration (Quevauviller, 2006): the Water Framework Directive (WFD, 2000/60/EC), the Groundwater Directive (GWD, 2006/118/EC), the Nitrates Directive (91/676/EEC), the Landfill Directive (99/31/EC) and the Directive on Environmental Quality Standards in the Field of Water Policy (EQSD, 2008/105/EC), recently amended by Directive 2013/39/EU.

The Water Framework Directive (WFD), and in particular the related Groundwater Directive on the protection of groundwater against pollution and deterioration, establish criteria for: (1) the assessment of good chemical status (2) the identification and reversal of environmentally significant pollutant trends; (3) preventing or limiting inputs of pollutants into groundwater.

In 2009, the EU funded a 5 year project named GENESIS (VII Framework Programme). The project, coordinated by the Norwegian Institute for Agricultural and Environmental Research (Bioforsk), involved 25 partners from 17 countries. The objective of the GENESIS was to integrate pre-existing and new scientific knowledge into the development of methods, concepts, and tools for better management of groundwater resources. One of the project's main tasks was to provide suggestions for the revision of the Groundwater Directive.

During the project experts with various background have been working together to develop the approach of a better integrated groundwater system management. Impacts of land use and climate change have been integrated with the conceptual models of groundwater systems emphasizing on the role of Groundwater Dependent Ecosystems (GDEs). Achievement of this goal has been possible by twining expertise and knowledge gained from case studies on different hydrogeological, land use, and socio-economic settings. As an outcome a range of methods has been presented for the future management of aquifers.

In July 2013, the European Commission opened a public consultation to provide input to the first review of the Directive Annexes. This consultation was intended for stakeholders, experts and practitioners in public authorities involved or interested in the implementation of the GWD. The expertise gained during the course of the GENESIS project implementation allowed for providing input to two of the four topics identified by the public consultation, in particular on updating the list of substances regulated at EU and national levels. The project addressed also the knowledge gaps related to the occurrence and risk assessment for different groundwater contaminants, including the emerging contaminants.

## Discussion

### The annex I should include the manmade substances and the annex II the natural ones

Annex I of the GWD contains Europe-wide environmental quality standards (EQS) for two types of pollutants: nitrates and active substances and relevant metabolites in pesticides. Annex II contains a minimum list of pollutants and indicators to be provided by Member States for the establishment of threshold values and identifies information on those pollutants and indicators which have been indicated as contributing to the characterization of bodies or groups of bodies of groundwater as being at risk. Article 10 of the GWD obliges the Commission to review Annexes I and II of the Directive every 6 years and to come forward with legislative proposals, if appropriate.

The Annex I set the EQSs according to the regulations being ain force at that time; in particular the nitrate standard was established based on epidemiological studies (WHO, 2011) and the pesticides standard took into account the detection limit of the analytical equipment available at the beginning of the 90 s.

For naturally occurring substances the variability of the groundwater systems across the EU does not allow for the determination of common threshold values for the whole European territory, similarly like for nitrates and pesticides, which are regarded as substances of anthropogenic origin. For the naturally occurring substances, threshold values are based on the determination of the natural groundwater quality expressed as Natural Background Levels (NBLs). However, NBLs are highly variable depending on the geological environmental (e.g., karstic aquifers, presence of geothermal fields, mineral deposits, etc) and the location of the aquifer (e.g., coastal aquifers). Additionally, the existence of pristine areas where NBLs can be estimated is questionable. For these reasons the substances that require the NBL assessment are reported in Annex II.

In the Annex II list two man made substances are reported (Tetrachloroethylene and Trichloroethylene), probably because these substances were considered as originating from the point and not the diffuse contamination sources. In our opinion the scope of the Annex I should be limited to manmade substances for which it is possible to establish EQS and the Annex II should be reserved for the substances that require assessing of the NBLs.

### A common monitoring protocol is required

In 2010, the European Commission reviewed the threshold values established by Member States (European Commission, 2010a). Up to date, threshold values (TVs) have been reported across EU for 158 pollutants/indicators, either synthetic or naturally occurring (European Commission, 2009, 2010a). The range of values and the methodologies used show great diversity across Member States. Moreover for some pollutants/indicators included in the Annex II not all Member States (MS) have established the TVs (Table 1). Thus, those propositions cannot be considered as comparable and consistent. For this reason a unique methodology for TVs assessment should be proposed by the EU and adopted by Member States. Based on the type of substances the methodology should account for different geological settings resulting in different NBLs in groundwaters, and the various Environmental Quality Standards (EQS) which are set taking into account the various receptors (aquatic or terrestrial ecosystem or “groundwater itself”) and the various legitimate uses of groundwater. The methodology should also call for a common monitoring protocol across EU member states, in order maximize reliability of monitoring results.

**Table 1:** **Number of member states (MS) that have established threshold values (TV) for the substances included in annex II of the GWD (European Commission, 2010a)**.

**Pollutants/indicators**	**Number of MS (EU27) that established TV**
Ammonium	20
Ammonium (as nitrogen)	2
Arsenic	21
Cadmium	19
Chloride	22
Conductivity	14
Lead	20
Mercury	18
Sulphate	21
Sum of Trichloroethylene and Tetrachloroethylene	10
Tetrachloroethylene	10
Trichloroethylene	10

### The distinction between the point and diffuse source of contamination is questionable

The inclusion of contaminants coming from household or from industrial drivers in the Annex I is subjected to intense criticism on the basis that they are the point sources. However, there is a strong evidence that the aquifers contaminated by industrial and household urban contaminants at European scale can no longer be considered as affected only by point-type sources because the urban areas become intermixed with agricultural and natural areas, as a consequence of the effects of urban sprawl; and a high number and density of potential sources of pollution and already polluted hotspots, gives to this type of contamination a “diffuse source” pattern and no more a “point source” one.

More than a quarter of the European territory is nowadays directly affected by urban land use (European Environmental Agency, 2006); urban areas are becoming the typical landscape of many parts of Europe or, at least, the typical landscape of areas inhabited by the majority of Europeans. The urban sprawl is advancing at a very high rate and the urban areas in 2013 are much more extended all around the continent, with respect to 2000, when the Water Framework Directive was adopted. Urban sprawl can be considered as one of the most significant land-use transformation affecting Europe and is now rightly regarded as one of the major challenges facing urban Europe today (European Environmental Agency, 2006). In 2006, in the same year when the Groundwater Directive was adopted, the Environmental European Agency reported that the urban-area expansion rate of several eastern and western European countries had increased by over three times with respect to the growth rate of their population. Many recently sprawled zones are located in alluvial and/or coastal areas or within mountainous and/or foothill basins, frequently i the recharge areas of the aquifers historically used for drinking water supply. In some cases, these areas are inside or close to protection areas of wells or springs.

In the Northern Italy, one of the most populated area in Europe characterized by a typical mixed urban-agricultural landscape, Scalenghe et al. (2011) studied the influence of 150 years of land use on anthropogenic and natural carbon stocks in Emilia-Romagna region, that represents 16% of the 71,000 km^2^ of the Po River watershed. At the present time it is home to about 18 million inhabitants, with an average population density of 300 people / km^2^. This region produces 40% of the Italian gross domestic product, one-third of the national industrial and arable production and has half of the Italian animal husbandry. The Human Footprint, which globally is 28, in this area scores higher than 50 (Sanderson et al., 2003). Scalenghe et al. (2011) used the amount of carbon contained in concrete as an indicator of urbanization demonstrating that it has increased in their study area from about 2.5 Tg in the 1954 to 7.8 Tg in 2003. The maps produced in their study show that urban areas constitute a diffuse point source. The Corine Land Cover (CLC2000) reports that artificial surfaces cover 6% of the basin area. The river Po management plan (Autorità di bacino del fiume Po, 2010) shows that in the Po valley 1.3 waste water treatment plants per 100 km^2^ and 4.6 industrial point discharges per 100 km^2^ are present. Above the aquifer the density of industrial discharges rises to 6.8. This change in the land use causes the increase of non-point sources defined as improperly managed construction sites (Frumkin, 2002), former contaminated industrial sites under remediation (Callender and Rice, 2000; Van Metre and Mahler, 2010) and the sewage system network that is worldwide known as one of the most important source of contamination in urban areas (Nolan et al., 2002; Masetti et al., 2007). The new residential areas are also entering on peripheral pre-contaminated sites associated with industrial activities or landfills (Nijenhuis et al., 2013).

### The DPSIR framework requires better characterization of the household contamination

The European Union identified the Driver, Pressure, State, Impact, Response (DPSIR) framework as the analytical framework for reaching the goal of achieving good groundwater status (European Commission, 2003). DPSIR has been used for identifying impacts and pressures on groundwater resources in order to attain the goal of good status across Europe. Under this approach, a “Driver” is an anthropogenic activity that can affect the environment; a “Pressure” is the direct effect of a Driver; and an “Impact” is the environmental effect of a Pressure. “State” is the environmental condition resulting from the Pressure and ‘Responses’ are the measures taken to improve the State. DPSIR coupled with the groundwater characteristics has been used to develop conceptual models and to establish local monitoring programmes. Conceptual models are a means of describing and optionally quantifying systems, processes and their interactions (European Commission, 2010b) and are developed to different incremental degrees of complexity.

The Common Implementation Strategy identified households, industry, agriculture, forestry, mines and quarries, dump and storage sites, water abstraction and flow enhancement as the Drivers that can affect the status of groundwater.

The analysis of the pollutants and of the indicators within DPSIR reveals that pressures coming from agriculture are monitored by at least three classes of compounds (trace elements, pesticides, and nutrients). Contaminants coming from households and industry are represented in Annex II only by trace elements and by two substances belonging to crude oil contaminants. The new amended Groundwater Directive should require the monitoring of more indicators representative of industrial and household contamination. Annex 1 should include at least an indicator representative of the Industry Driver and an indicator representative of Household driver.

### Tetrachloroethylene and trichloroethylene can be candidate indicators of the urban sprawl

Urban sprawl phenomenon is a source of new groundwater pollutants coming from industrial and domestic sources: chemicals not previously included in national or international monitoring programmes (Reemtsma et al., 2008), and contaminants that have long occupied attention, are gaining new notoriety with the discovery of new aspects of their occurrence, fate or effects (Daughton, 2004). All those substances are labeled as emerging pollutants and they encompass antiseptics; abuse drugs, antioxidants; corrosion inhibitors, fragrances, insect repellents, flame retardants, gas propellants, plasticizers, pharmaceuticals, solvents, stain repellents, sterols, stimulants, sunscreens and surfactants (Balderacchi et al., 2013; Fatta-Kassinos and Michael, 2013).

Among those Emerging Contaminants, Tetrachloroethylene or Perchloroethylene (PCE) and Trichloroethylene (TCE) can be considered an excellent indicators of the groundwater pollution by multi-source diffuse type urban pollution because their environmental behavior and toxicity have been thoroughly studied. We suggest removing those compound from annex II and including them in the EQS list of the improved annex I for three reasons:

#### Chlorinated solvents, like PCE and TCE, are the most prevalent organic contaminants found in groundwater (Stroo et al., 2003)

Chlorinated aliphatic solvents are a large family of compounds that are widely used in several industries all over Europe. Due to improper use and disposal practices, these solvents (mainly PCE and TCE) are common organic contaminants of soil and groundwater (Kao and Lei, 2000; Rivett and Feenstra, 2005). They have a peculiar ability to infiltrate rapidly into the subsurface, causing soil and groundwater pollution (Kueper et al., 2003; Cortés et al., 2011). There are several thousand of PCE and TCE impacted sites throughout North America, continental Europe and other industrialized areas of the world (Hunkeler and Aravena, 2000; Kueper et al., 2003; Parker et al., 2003; Cortés et al., 2011). Many of these sites are affected by releases of DNAPL that took place since the first half of the 20th century (Kueper et al., 2003).

#### PCE and TCE have features that favor their persistence and areal diffusion in groundwater

They are typically mobile and recalcitrant (Guilbeaut et al., 2005; Rivett and Feenstra, 2005) and they originate, at the source, as immiscible liquids: at many PCE and TCE spill sites, residual amounts of these compounds persist in a pure liquid phase, commonly referred to as dense non-aqueous-phase liquids (DNAPLs), within pore spaces or fractures (U.S. EPA., 1992; Feenstra et al., 1996; Kao and Lei, 2000). Ground water flowing through the DNAPL zones dissolves them, generating plumes that commonly achieve exceptionally large sizes (Schwille, 1984, 1988; Mackay and Cherry, 1989). PCE and TCE may decompose into even more hazardous pollutants. Where the unfavorable environmental conditions occur, the persistence of these compounds in the hydrogeological system can lead to reductive microbial dechlorination of PCE and TCE to dichloroethene (DCE) isomers and vinyl chloride (VC) (Vogel and McCarty, 1985; Barrio-Lage et al., 1987), which are well known carcinogenic compounds. Moreoer, they can cross low permeability geological layers that do not act as protective barriers as they do for groundwater. Solvent DNAPLs can penetrate into or through most types of aquitards, even those with very low bulk hydraulic conductivity, due to naturaly occurring preferential pathways (Parker et al., 2004). Resultingly, not only phreatic aquifers but also the confined ones are highly vulnerable toward PCE and TCE contamination. They can closely interact with low-permeability deposits, especially those having a significant organic matter content, where they can be trapped and subsequently released into the aquifers (Chapman et al., 2012) in such a manner that these deposits become a econdary source of pollution persisting for times estimated at up to hundreds of years (Chapman and Parker, 2005). Consequently, low permeability deposits can release to groundwater significant amounts of pollutants resulting through such back diffusion in their dangerous concentrations, (Parker et al., 2004). The occurrence and the persistence of these processes corroborate the opinion that they should be considered as representative of non-point pollution sources too. Remediation of these pollutants can be very difficult to conduct. Experience from the past 20 years has demonstrated that DNAPL sites are difficult to investigate and challenging to remediate. Interest in strategies for source removal has increased since the mid-1990s. Innovative technologies were developed and marketed to overcome the perceived technical impracticability of source treatment (Kueper et al., 2003).Therefore, given that it is often not possible to locate and remove the residual PCE and TCE, remediation should focus on preventing further migration of dissolved contamination (Kao and Lei, 2000).

#### PCE and TCE have been detected as ubiquitous in extensive areas of european aquifers

Many urban areas all around Europe observe a widespread pattern of PCE and TCE occurrence in groundwater Very often plumes, coming from single point-sources, intermingle and coalesce between each other revealing a diffuse pattern of pollution. Chlorinated solvents typically cause a persistent environmental contamination, which, in most cases, started decades ago (Cortés et al., 2011).

In their studies in the UK, Longstaff et al. (1992) found that low-level contamination is often widespread, with “hotspots” of higher concentrations. “Hotspots” of higher level groundwater contamination are often associated with sites where solvents were used, although obvious sources of solvent contamination could not be found for all “hotspots.” Moreover, several studies from over the whole Europe report cases in which PCE and TCE contamination is widespread on a large area, even if it originated from point sources (Italy: Cavellero et al., 1985; CNIC, 2010; Nijenhuis et al., 2013; Netherlands: Zoetmann et al., 1981; Germany: McCann and Appleton, 1993; Spain: Cortés et al., 2011; UK: Rivett et al., 1990). A recent investigation, conducted in Campania region nearby Naples (Southern Italy), has put in evidence that, inside a mainly agricultural-used land, due to the occurrence of multi-sources illegal dumps, PCE is the most widespread contaminant (CNIC, 2010).

### Should we consider other emerging substances?

Until now, water quality legislation has not systematically dealt with emerging pollutants for several reasons, including a lack of knowledge on contaminant sources and pathways, the properties and effects of substances and analytical detection techniques. Recently, scientific interest is given to organics waste water contaminants (OWCs), which are all the substances that reach waste water treatment plant, from veterinary antibiotics to industrial contaminants. Critical opinions against the monitoring of other emerging pollutants arise because of the high cost of the monitoring and the poor knowledge about those substances. However the study of those “emerging substances” is required for the progress of the science and for giving to the citizen a more safe environment. Because of this safety demand and the lack of conceptual models for emerging pollutants in groundwater, policy-makers and scientists are cooperating for the creation of an initial groundwater emerging pollutant priority list. The directive 2013/39/EU introduces a watch list of substances that will be completed in 2014 and that now includes Diclofenac, 17-beta-estradiol and 17-alpha-ethinylestradiol.

The classification of emerging pollutants according to the source of contamination or the toxicity is effective and pragmatic but does not consider the interaction of the substances with the soil that in the case of groundwater contamination is crucial and therefore the development of the conceptual model is expected. The selection of those substances has to be carried out considering two main drivers pressures:

1. The diffuse contamination coming from the agriculture driver. In this case, the hydrogeological conceptual models are already available and therefore biogeochemical models can be developed easily. Veterinary pharmaceuticals are good candidates for the inclusion in the monitoring plans because they are pharmaceuticals widely used: Kools et al. (2008) estimated that more than 5000 t of antibiotics and 5 t of hormones are employed in the European meat production. The substances are generally known and not patented because they have been on the market since long time. They contaminate the manure and slurry and are distributed during the agricultural production assuming diffuse contamination path.
2. The quasi “point source” contamination coming from household. In view of the scarce knowledge of the contamination pathways, the first attempts will have to focus on simplified pathways: from households to WWTPs to rivers and to groundwater; from biosolids and graywater to soils and groundwater. It is also required to collect information on monitoring point construction details, hydrological settings, aquifer type, understanding of recharge sources and patterns, local groundwater flow patterns and regimes, abstraction impacts, residence times and groundwater age distributions (European Commission, 2007). Environmental tracers can provide valuable information on natural attenuation of dissolved organic contaminants in groundwater systems. Isotope tracers constitute an important and widely used tool in studies on the environmental fate of biodegradable organic contaminants (Hunkeler et al., 2008; Aelion et al., 2009), providing evidence for and insights into mechanisms of microbial decomposition.

## Conclusions

Nowadays more than a quarter of the European territory has been directly affected by urban land use and the changes in the society and in particular the urban sprawl phenomena is also changing the current conceptual models based on the DPSIR framework. The scientific progress is providing evidence, that contamination pathways, previosuly linked to point sourcesare now considered as originating from diffuse sources. New environmental quality standards have to be defined in addition to nitrates and pesticides for protecting the groundwater quality at the European scale. PCE and TCE are increasingly destined to be considered as indicators of a widespread source of contamination, rather than a point-source phenomenon and therefore they can be candidate to be the best indicator of the groundwater pollution by multi-source diffuse type urban pollution of groundwater.

Other emerging pollutants are attracting the interest of scientists and policy makers. The monitoring of those substances is a necessary step in the creation of conceptual models for the improvement of the DPIRS framework. The veterinarian pharmaceuticals framed within the driver agriculture and the pressures associated to this driver can be an excellent starting point because diffuse contamination agricultural conceptual models are already developed and these models can help to understand the behavior of pharmaceuticals into the environment.

### Conflict of interest statement

The authors declare that the research was conducted in the absence of any commercial or financial relationships that could be construed as a potential conflict of interest.

## References

[B49] Aelion C. M. Höhener P. Hunkeler D. Aravena R. (2009). Environmental Isotopes in Biodegradation and Bioremediation. Boca Raton, FL: CRC Press

[B1] Autorità di bacino del fiume Po. (2010). Piano di Gestione del Distretto Idrografico del Fiume Po. Available online at: http://www.adbpo.it/on-multi/ADBPO/Home/PianodiGestioneepartecipazionepubblica/PianodiGestionedelDistrettoidrograficodelfiumePo.html (Accessed 19 March, 2014).

[B2] Balderacchi M. Benoit P. Cambier P. Eklo O. M. Gargini A. Gemitzi A. (2013). Groundwater pollution and quality monitoring approaches at the European level. Crit. Rev. Environ. Sci. Technol. 438, 312–318 10.1080/10643389.2011.604259

[B3] Barrio-Lage G. Parsons F. Z. Nassar R. S. (1987). Kinetics of the depletion of trichloroethene. Environ. Sci. Technol. 21, 366–370 10.1021/es00158a005 22280176

[B4] Callender E. Rice K. C. (2000). The urban environmental gradient: anthropogenic influences on the spatial and temporal distributions of lead and zinc in sediments. Environ. Sci. Technol. 34, 232–238 10.1021/es990380s

[B5] Cavellero A. Corradi C. De Felice G. Grassi P. (1985). Underground water pollution in Milan by industrial chlorinated organic compounds, in Effects of Land Use Upon Fresh Waters, eds de J. F. Solbe L. G. (Chichester: Ellis Harwood), 65–84

[B6] Chapman S. W. Parker B. L. (2005). Plume persistence due to aquitard back diffusion following dense nonaqueous phase liquid source removal or isolation. Water Resour. Res. 41, 1–16 10.1029/2005WR004224

[B7] Chapman S. W. Parker B. L. Sale T. C. Doner L. A. (2012). Testing high resolution numerical models for analysis of contaminant storage and release from low permeability zones. J. Contam. Hydrol. 136–137, 106–116 10.1016/j.jconhyd.2012.04.006 22750557

[B8] CNIC – Naval Support Activity Nalpes. (2010). Final Naples Public Health Evaluation. (Final Report - Vol I: Tetra Tech, 2010; Vol II: Pioneer, 2010; Vol III: Navy and Marine Corps Public Health Center, 2010). Available online at: http://www.cnic.navy.mil

[B9] Cortés A. Puigserver D. Carmona J. M. Viladevall M. (2011). Biological remediation approach involving soils and groundwaters polluted with chlorinated solvents in a Mediterranean context, in Recent Advances in Pharmaceutical Sciences, ed Muñoz-Torrero D. (Trivandrum: Transworld Research Network), 223–246 ISBN: 978-81-7895-528-5.

[B10] Daughton C. G. (2004). Non-regulated water contaminants: emerging research. Environ. Impact Assess. Rev. 24, 711–732 10.1016/j.eiar.2004.06.00322985674

[B11] European Commission. (2003). Analysis of Pressures and Impacts - Guidance Document No 3. Luxembourg: Office for Official Publications of the European Communities

[B53] European Commission. (2007). Guidance Document No. 15 - Guidance on Groundwater Monitoring. Luxembourg: Office for Official publications on the European Communities

[B12] European Commission. (2009). Guidance on Groundwater Status and Trend Assessment, Guidance Document no 18. Technical Report 2009. Luxembourg: European Communities ISBN: 978-92-79-11374-1.

[B13] European Commission. (2010a). Commission Staff Working Document accompanying the Report from the Commission in Accordance with Article 3.7 of the Groundwater Directive 2006/118/EC on the Establishment of Groundwater Threshold Values, SEC(2010) 166 Final. Bruxelles

[B14] European Commission. (2010b). Guidance Document No. 26 - Guidance on Risk Assessment and the Use of Conceptual Model for Groundwater. Luxembourg: Office for Official publications on the European Communities

[B15] European Environmental Agency. (2006). Urban Sprawl in Europe. The Ignored Challenge Report No 10/2006. Luxembourg: Office for Official Publications of the European Communities

[B17] Fatta-Kassinos D. Michael C. (2013). Wastewater reuse applications and contaminants of emerging concern. Environ. Sci. Poll. Res. 20, 3493–3495 10.1007/s11356-013-1699-5 23613205

[B18] Feenstra S. Cherry J. A. Parker B. L. (1996). Conceptual models for the behavior of DNAPLs in the subsurface, in Dense Chlorinated Solvents and Other DNAPLs in Groundwater: History, Behavior and Remediation, eds Pankow J. F. Cherry J. A. (Rockwood, ON: Waterloo Educational Services), 53–88

[B19] Frumkin H. (2002). Urban sprawl and public health. Public Health Rep. 117, 201–217 10.1016/S0033-3549(04)50155-3 12432132 PMC1497432

[B21] Guilbeaut M. A. Parker B. L. Cherry J. A. (2005). Mass and Flux distribution from DNAPL zones in sandy aquifers. Ground Water 43, 70–86 10.1111/j.1745-6584.2005.tb02287.x 15726926

[B22] Hunkeler D. Aravena R. (2000). Determination of compound-specific carbon isotope ratios of chlorinated Methanes, Ethanes, and Ethenes in aqueous samples. Environ. Sci. Technol. 34, 2839–2844 10.1021/es991178s

[B48] Hunkeler D. Meckenstock R. U. Sherwood Lollar B. Schmidt T. C. Wilson J. T. (2008). A Guide for Assessing Biodegradation and Source Identification of Organic Ground Water Contaminants using Compound Specific Isotope Analysis (CSIA). Ada, OK: USEPA EPA 600/R-08/148.

[B23] Kao C. M. Lei S. E. (2000). Using a peat bio-barrier to remediate PCE/TCE contaminated aquifers. Water Res. 34, 835–845 10.1016/S0043-1354(99)00213-4

[B24] Kools S. A. E. Moltmann J. F. Knacker T. (2008). Estimating the use of veterinary medicines in the European union. Regul. Toxicol. Pharmacol. 50, 59–65 10.1016/j.yrtph.2007.06.003 18006198

[B25] Kueper B. H. Wealthall G. P. Smith J. W. N. Leharne S. A. Lerner D. N. (2003). An Illustrated Handbook of DNAPL Transport and Fate in the Subsurface. Environment Agency, Almondsbury, Bristol: R&D Publication 113, 67. ISBN: 1844320669.

[B26] Longstaff S. L. Aldous P. J. Clark L. Flavin R. J. Partington J. (1992). Contamination of the chalk aquifer by chlorinated solvents: a case study of the Luton and Dunstable area. J. Inst. Water Environ. Manag. 6, 541–550 10.1111/j.1747-6593.1992.tb00789.x

[B27] Mackay D. M. Cherry J. A. (1989). Groundwater contamination: pump-and-treat remediation. Environ. Sci. Technol. 23, 630–636 10.1021/es00064a001

[B28] Masetti M. Poli S. Sterlacchini S. (2007). The use of the weights-of-evidence modeling technique to estimate the vulnerability of groundwater to nitrate contamination. Nat. Resour. Res. 16, 109–119 10.1007/s11053-007-9045-6

[B29] McCann B. Appleton B. (1993). European Water: Meeting the Supply Challenges. A Financial Times Management Report. London: Financial Times Business Information

[B30] Nijenhuis I. Schmidt M. Pellegatti E. Paramatti E. Richnow H. H. Gargini A. (2013). A stable isotope approach for source apportionment of chlorinated ethene plumes at a complex multi-contamination events urban site. J. Contam. Hydrol. 153, 92–105 10.1016/j.jconhyd.2013.06.004 24077332

[B31] Nolan B. T. Hitt K. J. Ruddy B. C. (2002). Probability of nitrate contamination of recently recharged groundwaters in the conterminous United States. Environ. Sci. Technol. 36, 2138–2145 10.1021/es0113854 12038822

[B32] Parker B. L. Cherry J. A. Chapman S. W. (2004). Field study of TCE diffusion profiles below DNAPL to access aquitard integrity. J. Contam. Hydrol. 74, 197–230 10.1016/j.jconhyd.2004.02.011 15358493

[B33] Parker B. L. Cherry J. A. Chapman S. W. Guilbeault M. A. (2003). Review and analysis of chlorinated solvent dense non-aqueous phase liquid distributions in five sandy aquifers. Vadose Zone J. 2, 116–137 10.2136/vzj2003.0116

[B34] Quevauviller P. (2006): The progress state of European implementation of the WFD. Houille Blanche-Revue Internationale De L'Eau 4, 55–57 10.1051/lhb:200604008

[B35] Reemtsma T. García-López M. Rodríguez I. Quintana J. B. Rodil R. (2008). Organophosphorus flame retardants and plasticizers in water and air I. Occurrence and fate. TrAC Trends Anal. Chem. 27, 727–737 10.1016/j.trac.2008.07.002

[B36] Rivett M. O. Feenstra S. (2005). Dissolution of an emplaced source of DNAPL in a natural aquifer setting. Environ. Sci. Technol. 39, 447–455 10.1021/es040016f 15707043

[B47] Rivett M. O. Lerner D. N. Lloyd J. W. (1990). Chlorinated solvents in UK aquifers. Water Environ. J. 4, 242–250 10.1111/j.1747-6593.1990.tb01385.x

[B37] Sanderson E. W. Jaiteh M. Levy M. A. Redford K. H. Wannebo A. V. Woolmer G. (2003). The human footprint and the last of the wild. BioScience 52, 891–904 10.1641/0006-3568(2002)052[0891:THFATL]2.0.CO;2

[B38] Scalenghe R. Malucelli F. Ungaro F. Perazzone L. Filippi N. Edwards A. C. (2011). Influence of 150 years of land use on anthropogenic and natural carbon stocks in Emilia-Romagna region (Italy). Environ. Sci. Technol. 45, 5112–5117 10.1021/es1039437 21609007

[B39] Schwille F. (1984). Migration of organic fluids immiscible with water in the unsaturated zone, in: Pollutants in Porous Media: The Unsaturated Zone Between Soil Surface and Groundwater, eds Yaron B. Dagan G. Goldschmidt J. (Berlin: Springer-Verlag), 27–48 10.1007/978-3-642-69585-8_4

[B40] Schwille F. (1988). Dense Chlorinated Solvents in Porous and Fractured Media: Model Experiments. (Transl. J. F. Pankow) Chelsea, Michigan: Lewis Publishers

[B41] Stroo H. F. Unger M. Ward C. H. Kavanaugh M. C. Vogel C. Leeson A. (2003). Chlorinated solvent source zones. Environ. Sci. Technol. 37, 224A–230A 10.1021/es032488k 12831011

[B42] U.S. EPA. (1992). Dense Nonaqueous Phase Liquids—A Workshop Summary. Dallas, TX. 17–18 April 1991. EPA/600/R-92/030. Robert S. Kerr Environmental Research Laboratory, Ada, OK

[B43] Van Metre P. C. Mahler B. J. (2010). Contribution of PAHs from coal-tar pavement sealcoat and other sources to 40 U.S. lakes. Sci. Total Environ. 15, 409 10.1016/j.scitotenv.2010.08.014 21112613

[B44] Vogel T. M. McCarty P. L. (1985). Biotransformation of tetrachloroethylene to trichloroethylene, Dichloroethylene, Vinyl chloride and carbon dioxide under methanogenic conditions. Appl. Environ. Microbiol. 49, 1080–1083 3923927 10.1128/aem.49.5.1080-1083.1985 PMC238509

[B45] WHO. (2011). Nitrate and Nitrite in Drinking-Water. Background Document for Development of WHO Guidelines for Drinking-water Quality. Available online at: WHO/SDE/WSH/07.01/16/Rev/1

[B46] Zoetmann B. C. F. De Greff E. Brinkmann F. J. J. (1981). Persistency of organic contaminant in groundwater lessons from soil pollution incidents in The Netherlands. Sci. Total Environ. 21, 41–46

